# Cocaine diminishes functional network robustness and destabilizes the energy landscape of neuronal activity in the medial prefrontal cortex

**DOI:** 10.1093/pnasnexus/pgae092

**Published:** 2024-03-06

**Authors:** Ahmad Borzou, Sierra N Miller, Jonathan D Hommel, J M Schwarz

**Affiliations:** Department of Physics and BioInspired Institute, Syracuse University, Syracuse, NY 13244, USA; CompuFlair, Houston, TX 77064, USA; Department of Pharmacology and Toxicology, Center for Addiction Sciences and Therapeutics, University of Texas Medical Branch, Galveston, TX 77555, USA; Department of Pharmacology and Toxicology, Center for Addiction Sciences and Therapeutics, University of Texas Medical Branch, Galveston, TX 77555, USA; Department of Physics and BioInspired Institute, Syracuse University, Syracuse, NY 13244, USA; Indian Creek Farm, Ithaca, NY 14850, USA

## Abstract

We present analysis of neuronal activity recordings from a subset of neurons in the medial prefrontal cortex of rats before and after the administration of cocaine. Using an underlying modern Hopfield model as a description for the neuronal network, combined with a machine learning approach, we compute the underlying functional connectivity of the neuronal network. We find that the functional connectivity changes after the administration of cocaine with both functional-excitatory and functional-inhibitory neurons being affected. Using conventional network analysis, we find that the diameter of the graph, or the shortest length between the two most distant nodes, increases with cocaine, suggesting that the neuronal network is less robust. We also find that the betweenness centrality scores for several of the functional-excitatory and functional-inhibitory neurons decrease significantly, while other scores remain essentially unchanged, to also suggest that the neuronal network is less robust. Finally, we study the distribution of neuronal activity and relate it to energy to find that cocaine drives the neuronal network towards destabilization in the energy landscape of neuronal activation. While this destabilization is presumably temporary given one administration of cocaine, perhaps this initial destabilization indicates a transition towards a new stable state with repeated cocaine administration. However, such analyses are useful more generally to understand how neuronal networks respond to perturbations.

**Significance Statemen**

Cocaine dependence affects the brain in various ways, including altering prefrontal cortex activity. The researchers construct a unique computational method by combining in vivo calcium imaging, the modern Hopfield model, and machine learning techniques to examine the effects of cocaine on the brain’s neuronal network in the medial prefrontal cortex of rats. They use this method to identify changes in network functionality following drug administration. Their findings indicate that cocaine disrupts network robustness and leads to decreased network stability, as demonstrated by changes in functional neuronal network characteristics and in the energy landscape for neuronal activity. These alterations contribute to our understanding of the initiation of cocaine dependence. This study therefore provides crucial insights into the neurobiological underpinnings of substance abuse, potentially informing better prevention strategies and therapeutic interventions.

## Introduction

The intricacies of a brain’s neuronal network drive its functionality. The characterization of such a network spans multiple scales ranging from the scale of genes to the scale of the whole brain. For instance, specific genes have been identified as playing a role in brain size and shape and, more recently, in long-range connectivity (1–6). At the brain scale, on other hand, there exists the nearly universal finding of a small-world network architecture in which most nodes can be reached by traversing some number of edges (7, 8). Others focus on particular neuronal circuits at the mesoscale to decode a specific functionality. For instance, it was recently revealed how a neuronal circuit involved in fruit fly navigation empowers a fly to perform vector addition (9). How such findings at the different scales can be integrated into one, more complete picture to understand the structure–function relationship in the brain is a current avenue of investigation (10, 11).

At the mesoscale, in brains with smaller numbers of neurons, such as the *Caenorhabditis elegans* or the fruit fly, determining neuronal circuits is feasible. In brains with larger numbers of neurons, determining such neuronal circuits is not as feasible. And yet, with the advent of new imaging techniques in freely moving animals, such as rodents, to record *individual* neurons deep inside the brain (12), one is compelled to assess the functional contribution of a collection of individual neurons forming a subnetwork. Here, we will study such a subnetwork and characterize its response to a particular perturbation. Our perturbation of choice is the impact of cocaine on a neuronal subnetwork of the medial prefrontal cortex.

Cocaine primarily impacts the brain’s limbic system by targeting dopaminergic pathways and increasing dopamine release (13, 14). The limbic system is a network of multiple brain regions including the prefrontal cortex and the nucleus accumbens, which are brain regions closely associated with motivated behavior and substance use disorders. The prefrontal cortex acts in a top-down manner to inhibit the nucleus accumbens and suppress motivated behavior (15). Therefore, stable function of the prefrontal cortex is critical to suppressing the abberant motivated behavior associated with substance dependence.

And so while the interconnectedness of the brain’s limbic, or emotional, system is key to understanding the effects of cocaine on the brain, here we focus on the medial prefrontal cortex. At the scale of the prefrontal cortex, there is a reduction in the prefrontal cortex volume in cocaine-dependent men that may help explain deficiencies in prefrontal cortex functionality (16). Moreover, electrophysiology has shown that the administration of cocaine decreases neuron activity in the medial prefrontal cortex (17). Additionally, from a neuronal network point of view, the likelihood of developing dependence is associated with significant hypoconnectivity in orbitofrontal and ventromedial prefrontal cortical–striatal circuits–pathways critically implicated in goal-directed decision-making (18). At smaller scales, there exists some evidence of neuronal remodeling at the single neuron level in the medial prefrontal cortex (19–21).

The impact of cocaine on the medial prefrontal cortex, in particular, has relied heavily on functional magnetic resonance imaging to understand the function of the region as a whole (16, 22, 23) or in vitro or ex vivo analyses of gene or electrophysiological profiles to understand single neuron alterations (19, 20, 24). Our approach, on the other hand, utilizes a combination of established and novel statistical mechanics methods to identify cocaine-induced alterations in the prefrontal cortex network at the mesoscale. Furthermore, we provide a new analytical tool for in vivo calcium imaging data from freely behaving rodents as well as show, for the first time to our knowledge, destabilizing effects within a statistical mechanics setting, of single-cocaine administration on the prefrontal cortex.

We will focus in on the mesoscale by recording from a subset of neurons in the medial prefrontal cortex of a living rodent before and after cocaine administration. In particular, we will model the neuronal subnetwork as a linearized version of a Hopfield model (25, 26) and use a machine learning approach to obtain its functional connectivity. Determining the functional connectivity map allows one to make powerful predictions about the dynamics of the network in terms of firing patterns in response to external inputs, for example. In addition, it also allows one to perform network analysis (27, 28) with the additional information of functional-excitatory and functional-inhibitory weights that are typically not known when analyzing a structural connectome from a network point of view (29). Many efforts are indeed underway to correlate the functional connectome with the structural connectome (30). On the other hand, even in the absence of such information, network analysis can identify subgraphs, which may correspond to a neuronal circuit with a particular function (31).

At the mesoscale, one can also toggle between dynamical approaches and statistical mechanics approaches, in which time-averaged information may be useful. Dynamical approaches with large groups of neurons are less feasible, at least in so far as obtaining the functional connectivity of the group. As for prior statistical mechanics approaches, much has been done to give rich, new insights into brain functionality such as Refs. (32–34). Moreover, statistical field theory approaches to neuronal networks also exist (35–37). We will use a mean field theory approach in which spatial degrees of freedom are averaged out to obtain new insights into a Landau–Ginsburg-like energy landscape for the effects of cocaine on the brain.

The outline of the manuscript is as follows. First, we detail the experiments. Then, we briefly outline both the theoretical framework of the Hopfield model, applied to a neuronal network, as well as a statistical mechanics approach. After presenting the results of the analysis from both approaches, we conclude with a discussion of the impact of our results.

## Experiments

### Animals

Male Sprague Dawley rats (n=3) (Harlan Inc, Houston, TX, USA) aged 10 weeks were initially pair housed and maintained on a diet of standard rat chow (Tekland Mouse/Rat Diet 7912, Harlan Laboratories, Inc., Indianapolis, IN, USA) ad libitum in their home cage. The colony was maintained on a standard 12-h light cycle (6:00–18:00), 71 °F with relative humidity of 30–50%. All animal use was carried out in accordance with the Guide for the Care and Use of Laboratory Animals and with the approval of the University of Texas Medical Branch Institutional Animal Care and Use Committee.

### Virus injection

Following a 1-week habituation period, rats were anesthetized using 1–5% isoflurane and placed in a stereotaxic surgical apparatus (Kopf, Tujunga, CA, USA). Each animal received a unilateral injection of 1.0 μL AAV1.Camk2a.GCaMP6m.WPRE.SV40 (Inscopix, Mountain View, CA, USA) at a rate of 0.2 μL per minute in the right hemisphere medial prefrontal cortex (AP + 3.0 mm, ML + 0.5 mm, DV −4.2 mm). The syringe remained in place for 5 min following completion of injection before being removed and the wound was closed. Rats were then placed in a clean cage on a heating pad and followed postsurgically to ensure recovery. Animals were habituated to daily handling following surgery.

### Gradient-index lens implantation

Four to five weeks after virus injection, rats were anesthetized using a 1–5% isoflurane. Three stainless steel surgical screws (one per skull plate) were inserted to provide stability for the lens. After screws were placed, rats underwent stereotaxic implantation of the ProView integrated gradient-index (GRIN) lens (0.6mm×7.3mm, Inscopix) (AP + 3.0, ML +0.5 mm, DV −4.00 mm) in the medial prefrontal cortex. Excess space between the craniotomy and the base of the lens was filled with adhesive cement (Metabond Quick! Adhesive Cement System, C&B), and then the lens was secured to the skull with dental cement (ACRAWELD Repair Resin, Henry Schein, Melville, NY, USA). A baseplate cover (Inscopix) was installed to protect each lens until imaging. Wound clips were applied, and the animals were placed in a clean cage on a heating pad and monitored postsurgically to ensure recovery. Following GRIN lens implantation, all animals were single-housed to protect the implant and were handled daily. Postmortem histology was done to verify targeting.

### Cocaine administration and calcium data acquisition

Once animals had recovered from lens implantation surgery (>3 weeks), they were habituated to light restraint and attachment of the miniscope (Inscopix). The miniscope was mounted and animals were placed in the testing chamber for recording at least three times prior to testing. The testing chamber was a 40 cm × 40 cm clear, Plexiglass container with cornbob bedding. During these recording sessions, field of view and imaging settings were optimized for each animal. All recordings were taken at 10 frames per second. On testing day, animals were given 5 min following miniscope attachment to acclimate to the chamber before recording began. Imaging for each animal was optimized, with gains 2.0–3.5 and LED power 0.5–1.3. Focal depths for each animal were chosen to capture the maximum number of clearly defined neurons in a single plane of recording. Thirty minutes of baseline calcium activity was recorded, followed by a 5-min pause during which animals received a single intraperitoneal injection of 15 mg/kg cocaine (National Institute of Drug Abuse, Research Triangle Park, NC, USA) in sterile 0.9% NaCl before being returned to the chamber. Thirty minutes of postadministration calcium data was recorded. Then the session was ended, and the animal was returned to its home cage.

### Calcium activity trace extraction

**Fig. 1.**
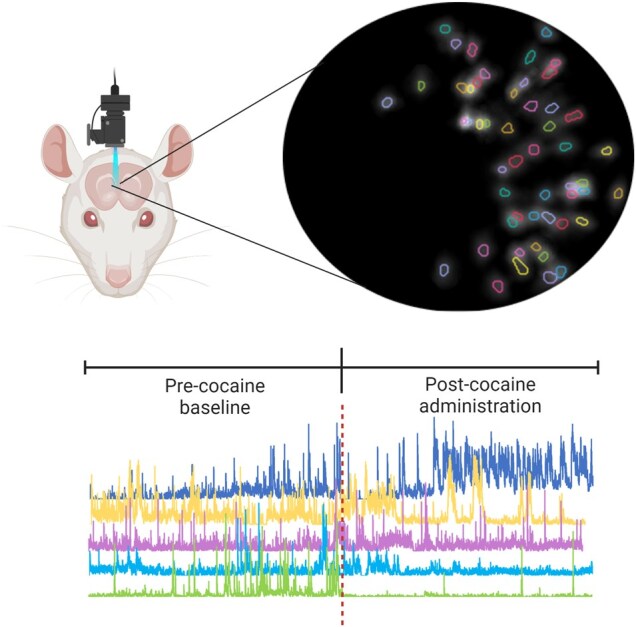
Schematic and data from the experiments. Top: Implanted microscope with image of neurons fluorescensing. Bottom: Representative relative calcium intensity traces time prior and post cocaine administration for several neurons; partially created from Biorender.com.

Initial calcium imaging data processing was done using the Inscopix Data Processing Software (Inscopix). Video processing steps were the same for every animal. Videos were spatially downsampled by a factor of four. Motion correction utilizing the “mean image” as a reference frame was done and CNMFe was used to identify neurons (38). Manual validation of CNMFe identified neurons was performed to ensure that all identified neurons used for analysis contained a clear baseline for the 30 min and well-defined, positive peaks of fluorescence. See Fig. 1. The calcium traces are, therefore, ΔF/F, where *F* denotes relative fluorescence intensity with respect to the baseline.

## Mathematical framework

In this section, we lay down the mathematical basis for the analysis we will use to determine the effects of cocaine on the medial prefrontal cortical network. We start from the modern Hopfield model of a neuronal network to write the governing differential equations of neurons’ firing states, and to construct the total energy of the system (26). We review how the classic Hopfield model can then be extracted from the modern version (25) as well as address its biological interpretation using a circuit analogy (39) for a simpler version. Next, we construct the statistical field theory of a neuronal network starting from the Hopfield model energy. Within the context of both approaches, we discuss how the firing behavior of the network can be influenced by external stimuli.

### Hopfield model of a neuronal network

The modern Hopfield Network (26) provides a mathematical description of the activity of neurons in the brain, which account for functional features, such as memory. In this model, neurons that are categorized as the feature neurons are referred to by Latin indices and hidden neurons are referred to by Greek indices. The feature neurons are the ones observed in an experiment and the hidden neurons are the rest of neurons that actually exist in the brain’s region of interest. The two categories of neurons are linked by a matrix $\xi_{i\mu }$ that represents the synaptic connectivity. The currents of the two types of neurons are denoted by $V_{i}$ and $h_{\mu }$. The output currents of the feature and hidden neurons are denoted by $g_{i}$ and $f_{\mu }$ and are defined as


(1) $$\begin{array}{rl} \;f_{\mu } & =\frac{\partial L_{h}}{\partial h_{\mu }},\\ g_{i} & =\frac{\partial L_{V}}{\partial V_{i}}, \end{array}$$

where $L_{h}$ and $L_{V}$ are the Lagrangians that define the model. Therefore, $f_{\mu }$ and $g_{i}$ are the conjugate variable to the currents of hidden and feature/visible neurons, respectively.

The full theory is described by two differential equations given by


(2) $$\begin{array}{rl} \tau \frac{dV_{i}}{dt} & =\sum_{\mu }\xi_{i\mu }f_{\mu }-V_{i}+V_{i}^{\left(\mathrm{ext}.\right)},\\ \tau_{h}\frac{dh_{\mu }}{dt} & =\sum_{i}\xi_{i\mu }g_{i}-h_{\mu }+h_{\mu }^{\left(\mathrm{ext}.\right)}, \end{array}$$

where $V_{i}^{\left(\mathrm{ext}.\right)}$ and $h_{\mu }^{\left(\mathrm{ext}.\right)}$ represent external currents that do not come from the rest of neurons in the system, *τ* is a time constant, and the sums run over all the neurons. The Hamiltonian of the neuronal network that drives its time evolution is given by


(3) $$\begin{array}{rl} E & =\left[\sum_{i=1}\left(V_{i}-V_{i}^{\left(\mathrm{ext}.\right)}\right)g_{i}-L_{V}\right]\\ & \;+\left[\sum_{\mu =1}\left(h_{\mu }-h_{\mu }^{\left(\mathrm{ext}.\right)}\right)f_{\mu }-L_{h}\right]-\sum_{\mu ,i}\xi_{i\mu }f_{\mu }g_{i}. \end{array}$$

Assuming that (i) the hidden neurons’ activities are rather fast and (ii) they do not receive external currents, we can set $\tau_{h}=h_{\mu }^{\left(\mathrm{ext}.\right)}=0$ and solve Eq. 2 to remove the current of the hidden neurons in terms of the connectivity matrix and the output current of the feature neurons, or


(4) $$h_{\mu }=\sum_{i}\xi_{i\mu }g_{i}.$$

This solution to the hidden current can be substituted into $f_{\mu }$ in the first line of Eq. 2 such that the differential equation of the current of the feature neurons is self-contained.

At this point, we need to specify the form of the Lagrangian, and, therefore, the Hamiltonian, to remove $f_{\mu }$ in terms of $V_{i}$ and $\xi_{i\mu }$. We choose a very simple form, namely,


(5) $$f_{\mu }=\sum_{j}\xi_{\;j\mu }V_{j}.$$

This form leads to a simpler linear version of the classic Hopfield model (25). As we will observe in the next section, this simpler linear version, as opposed to piecewise linear, readily approximates the calcium intensity signal, which has a longer time constant than action potentials. Given the longer time constant, combined with experimental limitations, observing thresholding in individual neurons is difficult and so a simpler integrate-and-fire neuron model for $f_{\mu }$ is more relevant here (40).

**Fig. 2.**
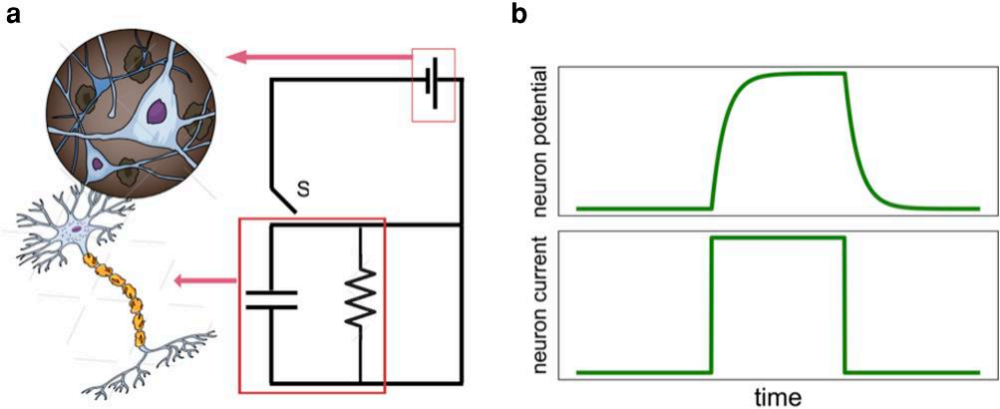
RC model of a single neuron. a) The battery represents the voltage from all other neurons as well as any external signal. The switch S represents charging and discharging cycles of the neuron. When a neuron consumes ATP to open the ion channels, S is connected to the battery. During the discharge phase S moves from the battery to the other side to form a closed self-circuit. In this phase regardless of the incoming signals from the other neurons, the neuron does not fire. b) The current and voltage cycle of a neuron after firing.

As has been done with the classic Hopfield model (25), as well as other neuronal network models (40), it is helpful to construct a circuit diagram of the neuronal network. We focus on one neuron in the network. More precisely, (i) a given neuron is replaced by a capacitor *C* with a resistor *R* in parallel, (ii) the remainder of neurons are replaced by a battery, and (iii) there exists a switch *S* that alternatively connects and disconnects the battery to the given neuron. Should the sum of functional-inhibitory and functional-excitatory input signals for a given neuron is above a threshold, the battery is connected to the given neuron to lead to a charging phase followed by discharging phase in which the neuron does not fire regardless of the input signal from the rest of neurons. When the given neuron is disconnected from the battery *S*, it forms a self-loop. The model is presented in Fig. 2 on the left. When the battery is connected, the electric current across the membrane of the neuron satisfies the following differential equation


(6) $$\begin{array}{rl} & \tau \frac{dV_{i}}{dt}=-V_{i}+V_{i}^{\left(b\right)},\\ & V_{i}^{\left(b\right)}\equiv \sum_{j}T_{ij}V_{j}+V_{i}^{\left(\mathrm{ext}.\right)}, \end{array}$$

where τ=RC with R being the resistance and C being the capacitance, the subscript *i* refers to the neuron that is modeled as an RC circuit, and $V_{i}^{\left(b\right)}$ is the current of the battery. Index *j* refers to all the rest of neurons while $T_{ij}$ represents the functional-excitatory or functional-inhibitory signal that neuron *j* sends to the neuron *i*. Using Eqs. 2 and 5, $T_{ij}$ has the following form


(7) $$T_{ij}=\sum_{\mu }\xi_{i\mu }\xi_{\;j\mu }.$$

It is this matrix, the functional connectivity matrix, that we will extract from the experimental data to forecast the neuronal network’s future, assuming there is no plasticity over some time scale.

Right after neuron *i* is fully charged, the discharge state begins when the switch S disconnects the battery and makes a self-loop where the neuron satisfies the following differential equation


(8) $$\tau \frac{dV_{i}}{dt}=-V_{i}.$$

Solving the two differential equations above, we readily find the current of neuron *i* in a full cycle whose solution is graphically depicted in right figure in Fig. 2 and given by


(9) $$V_{i}(t)=\left\{ \begin{array}{cc} V_{i}^{\left(b\right)}\left(1-e^{-t/\tau }\right), & 0<t<T\\ V_{i}^{\left(b\right)}e^{-t/\tau }, & T<t \end{array}\right.$$

where *T* refers to the time that the switch disconnects the battery.

### Statistical properties of the neuronal network

It is also interesting to look beyond the microscopic details of the system, in terms of a functional connectivity matrix, to quantify its macroscopic properties as well in a statistical sense. In other words, the emergent properties of the neuronal networks can also be understood through studying the macroscopic behavior of the system using techniques from statistical mechanics as has been done previously (32–36).

We now consider fluctuations of the neuronal subnetwork, due to the surrounding neurons and other smaller scale influences. As this living system is not in equilibrium, we invoke a nonequilibrium version of equilibrium statistical mechanics where $T_{\mathrm{eff}}$ is some effective temperature (41–43). Moreover, the unnormalized probability of occurrence of a state with energy *E*, after integrating out the hidden neurons in Eq. 3, is equal to $e^{-E}$, with the $k_{B}T_{\mathrm{eff}}=1$, as it maximizes the entropy of the system (44–47). Therefore, the partition function, i.e. the weighted sum over all the energy states reads


$$Z=N^{\prime }\prod_{i}\int dV_{i}\text{exp}(-E(V_{i})),$$

where $N^{\prime }$ is the normalization factor.

We will assume that the energy fluctuates around some local minimum $E_{0}$ such that


(10) $$E=E_{0}+\vec{J}\cdot \vec{V}+\frac{1}{2}\vec{V}\cdot G^{-1}\cdot \vec{V}+O\left(V^{3}\right)+E_{\mathrm{no}-V},$$

where $\vec{V}=(V_{1},V_{2},\ldots )$ encodes the fluctuations of the system, the external source to the system of neurons is represented by $\vec{J}$, the Greens’ function *G* is an unknown to be derived from data, and the rest of the terms that do not depend on *V* are denoted by $E_{\mathrm{no}-V}$. It is assumed that there exists at least one stable, local minimum. Of course, there may be multiple local minima that could complicate our analysis. The partition function can then be rewritten in the following form


(11) $$Z=N\int DV\;e^{-E^{\prime }},$$

where the new normalization factor N, the path integration ∫DV, and the energy $E^{\prime }$


(12) $$\begin{array}{rl} N & \equiv N^{\prime }\sum_{E_{\mathrm{no}-V}}e^{-E_{\mathrm{no}-V}},\\ \int DV & \equiv \prod_{i}\int dV_{i},\\ E^{\prime } & \equiv \frac{1}{2}\vec{V}\cdot G^{-1}\cdot \vec{V}-\vec{V}\cdot \vec{J}+O(V^{3}). \end{array}$$

In principle, we should be able to use the partition function in Eq. 11, together with $\vec{V}$, and study the collective behavior in this space. In practice, due to the high dimensionality of $\vec{V}$ and the typically small number of collected data points, we choose to define a different parameterization with lower number of features. In other words, let us use a mean field representation for the average of all the neurons firing, or neuronal activity, at a given time, i.e.


(13) $$m\equiv \frac{1}{N}\sum_{i=1}^{N}\frac{V_{i}}{V_{0}},$$

where $V_{0}$ is some reference voltage. Therefore, we reorganize the sum in the partition function in Eq. 11 and define a new energy E[m] such that


(14) $$\begin{array}{rl} Z & =N\int Dm\;\int DV{|}_{m}e^{-E^{\prime }[\vec{V}]}\\ & =N\int Dm\;e^{-E[m]}, \end{array}$$

where $\int DV{|}_{m}$ refers to a summation on all the possibilities in $\vec{V}$ space that are constrained to a fixed *m* as defined in Eq. 13. Also, ∫Dm refers to the summation over all the values of *m*. Finally, given the above expansion about a minimum, we consider a similar expansion in *m*, or


(15) $$E[m]=a_{0}+a_{1}m+a_{2}m^{2}+a_{3}m^{3}+a_{4}m^{4}+O(m^{5}),$$

where $a_{i}$ are constants to be determined by data. Since these parameters determine the collective behavior of the neuronal network in *m* space, or the mean field neuronal activity space, we would like to learn see their variations by changing the experimental condition. We will address this possibility in the Results section. Interestingly, there has been a study quantifying the mean activity of the system as a function of time using the Wilson–Cowan equations (48, 49) for neuronal activity as a starting point and then performing a similar expansion in the neuronal activity (37). Here, we investigate the distribution of the mean activity over time to assess its form. Moreover, as we will see, the data are well characterized by the expansion.

## Results

We now use the data collected from the medial prefrontal cortex before and after cocaine injection as described in Experiments section to find the free parameters of the mathematical model in Mathematical framework section. First, we extract the synaptic connectivity of the neurons for each of the two experimental conditions. Next, we use the statistical field theory approach to predict the firing behaviors of the networks of each experimental conditions when they receive planned external stimulus.

### Inferring the functional connectivity of the neuronal network from neuronal activity

Here, we derive the $T_{ij}$ connectivity matrices, and $V_{i}^{\left(\mathrm{ext}.\right)}$ in Eq. 6 by using a machine learning based model for each of the two datasets of Experiments section. From now on, we refer to the two experimental conditions as “before the injection” and “after the injection.” It should be noted that we use a CamKII promoter for GCaMP expression. While this promoter has been thought to be specific to excitatory glutamatergic neurons, multiple lines of study show this specificity is not present, particularly in cortical circuits (50, 51). As such, we make no assumptions about the biological identity of individual neurons and use functional-excitatory and functional-inhibitory to describe the directionality of the inferred correlations between neurons. Therefore, the same neuron can participate in both functional-excitatory and functional-inhibitory relationships within the overall network. Stated another way, a neuron’s calcium fluorescence may be positively or negatively correlated with the fluorescent intensity of other neurons in the network, which we describe as functional-excitatory or functional-inhibitory, respectively.

To remove background noise from each neuronal trace, each neuron, measurements of relative calcium intensity with a value smaller than a threshold are set to zero. We determine this threshold using the following method. We perform a sensitivity analysis by varying the relative calcium intensity threshold parameter. The procedure involves systematically changing this threshold parameter within the range of (0–20) and observing how these changes affect the coefficients and intercepts of the time-series linear regression model to be described below. For each threshold value, we select a random 70% subset of data, fit the time-series linear regression model to this subset, and store the model’s coefficients and intercepts. This process is repeated for each threshold value, generating a series of models. After all models are generated, we assess the stability of these models by comparing the coefficients and intercepts across successive threshold values. This comparison helps in understanding how sensitive the model parameters are to the changes in the threshold. The aim of this analysis is to identify a threshold that ensures model robustness, meaning the model parameters do not significantly change with small variations in this threshold. Fig. S2 shows the relative calcium ion intensity change of the first neuron in the set over the timing of the experiment. Fig. S3 shows the recorded relative intensities of the entire set of neurons and the applied selections prior and post cocaine administration.

To begin to analyze the functional connectivity of the neuronal network, we first time discretize Eq. 6 assuming that the RC time constant is of order the time increment δt such that


(16) $$V_{i}(t+\delta t)=\sum_{j}T_{ij}V_{j}(t)+V_{i}^{\left(\mathrm{ext}.\right)}(t).$$

In this equation, we set δt=0.1 s to be consistent with the frequency at which neuron activity is recorded, the time scale at which we explore the functional connectivity of the neurons. This equation has the same form as the formulation of the supervised auto-regression time-series forecasting machine learning method, where the value of a feature such as $V_{i}$ can be predicted using its values in the past. To perform the learning analysis, we prepare the predictor dataset *X* in a matrix form such that each component holds a value of $V_{i}$ in a way that the rows refer to the time of the feature in an ascending order and rows refer to each of the neurons. We also prepare the response variables *y* for each neuron separately. The latter is again the same $V_{i}$ values for the corresponding neuron but at one time step later. Having the *X*, and *y* variables, we use the LinearRegression model of Scikit-Learn package (52) in python to perform the regression and use the returned coefficients and intercept as the $T_{ij}$ and $V_{i}^{\left(\mathrm{ext}.\right)}$ of that neuron. We repeat the process for all the neurons.

For each of the experimental conditions, we use 75% of the dataset for training the model, i.e. estimating $T_{ij}$ and $V_{i}^{\left(\mathrm{ext}.\right)}$, and the remaining 25% for testing the quality of the extracted parameters. For the training part, we label the predicted value of Eq. 16 as ${\hat{V}}_{i}(t)$ and define the error function to be


(17) $$\text{Err}\left(T_{ij},V_{i}^{\left(\mathrm{ext}.\right)}\right)\equiv \frac{1}{N}\sum_{d=1}^{N}{\left({\hat{V}}_{i}^{\left(d\right)}(t)-V_{i}^{\left(d\right)}(t)\right)}^{2},$$

where *N* is the number of neurons, $V_{i}(t)$ is the observed value, and *d* runs over the data points in the training dataset. Our estimation for $T_{ij}$ and $V_{i}^{\left(\mathrm{ext}.\right)}$ are the solutions to the following equations


(18) $$\begin{array}{rl} & \frac{\partial \text{Err}}{\partial T_{mn}}=0,\\ & \frac{\partial \text{Err}}{\partial V_{k}^{\left(\mathrm{ext}.\right)}}=0. \end{array}$$

We separately perform the minimization in Eq. 18 for prior and post cocaine administration. The predictions of Eq. 16 are compared with their corresponding true values in Fig. S1. The figure indicates that the test error is small and our predictions are close to the true values in most of the times. Our estimations for $T_{ij}$ and $V_{i}^{\left(\mathrm{ext}.\right)}$ for before and after cocaine administration are the outputs of this minimization. In other words, the sum of inputs $V_{i}^{\left(\mathrm{ext}.\right)}$, from the hidden neurons, to the feature neurons is also estimated through our minimization. We find that these are negligible with respect to $T_{ij}$ matrices. The external inputs are shown in Fig. S4.

**Fig. 3.**
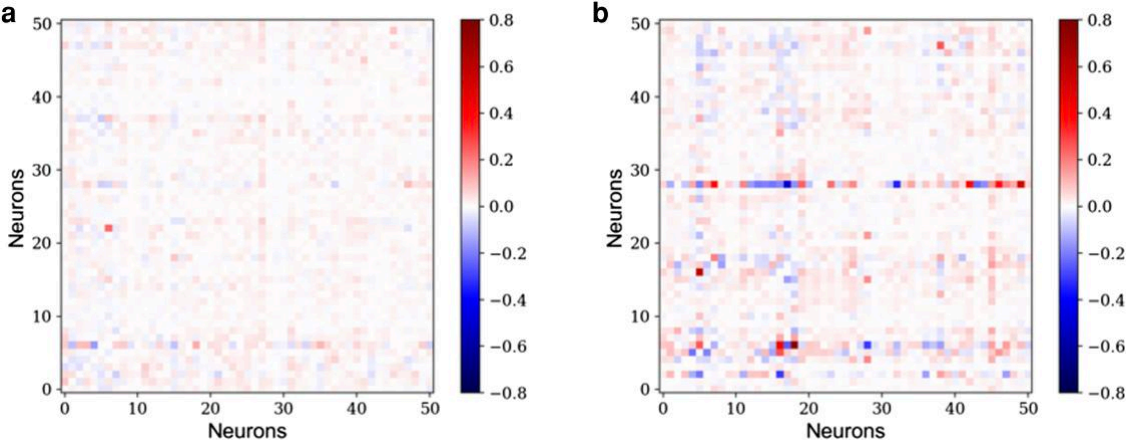
The functional connectivity matrix functional-inhibitory and functional-excitatory neurons prior a) and post b) cocaine administration. The $T_{ij}$ matrix is derived using a time series machine learning analysis of the calcium signaling in the neuronal network with negative weights representing functional-inhibitory connections and positive weights representing functional-excitatory connections.

**Fig. 4.**
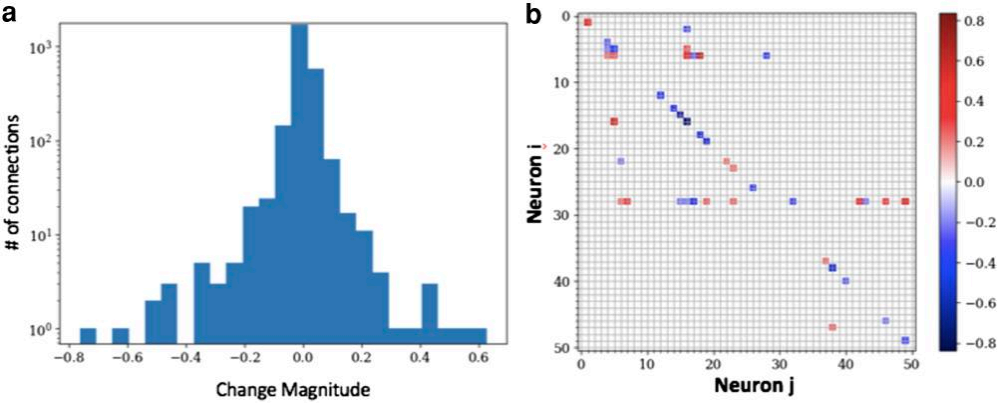
Changes in $T_{ij}$ to highlight the differences in the functional connectivity prior and post cocaine administration. a) The histogram demonstrates that there both increases and decreases in the weight connections. b) The changes in $T_{ij}$ prior and post cocaine.

We then use the resulting estimate of $T_{ij}$ to understand how cocaine administration may alter network function in the medial prefrontal cortex. We see a significant change between the connectivity matrices $T_{ij}$ of before and after cocaine administration. The two matrices are shown in Fig. 3, where each of the 51 neurons in the experiment are shown on both *x* and *y* axes. The functional-excitatory and functional-inhibitory neurons, i.e. the positive and negative components of $T_{ij}$, for both of the experimental conditions are shown in Fig. 3. A few conclusions can be readily drawn from the figure. First, the number of the functional-excitatory signals, shown by red colors, is close to two times the number of the functional-inhibitory signals, shown by the blue colors, in both of the experimental conditions. This result is in surprising agreement with biological observations of the actual synaptic connectivity (53, 54). Second, the connectivity matrices $T_{ij}$ are not symmetric, which is again in agreement with neuroscience expectations (55–57). Third, the interneuron signaling prior to the cocaine administration, when the neuronal network is in its “normal” state, is less significant than the signaling activities post cocaine administration. However, interestingly, we observe that the changes in decreasing the functional weights and increasing them are somewhat symmetric (see Fig. 4). This finding suggests that, at least at this scale of 50 neurons, the activity of the medial prefrontal cortex is modified beyond an overall decrease in activity, as reported in electrophysiology measurements on the scale of the brain region (16). Our analysis captures more subtle changes in functionality of the neuronal network. It should be mentioned that the functional connectivity matrix is symmetric with 50 dimensions (neurons). Hence, its unique components total 1,275. Additionally, the external voltage contribution introduces another 50 variables. Consequently, to resolve all unknowns, a minimum of 1,325 data points is required. This translates to an approximate need of at least 2.2 min of data, taken at 0.1 s intervals, for a comprehensive analysis, providing a rough estimate for the lower bound on the amount of data required.

To obtain further insight into the change in functional connectivity before and after cocaine administration, we performed conventional network analysis using the python package NetworkX. In NetworkX, the diameter of a graph is defined as the maximum eccentricity among all pairs of nodes in the graph. The eccentricity of a node is the maximum distance from that node to any other node in the graph. For weighted graphs, the distance between nodes is defined as the sum of the weights along the shortest path between them. The diameter function in NetworkX takes this into account when working with weighted graphs. Moreover, the betweenness centrality for a node *v* is defined as the fraction of all shortest paths, between all possible node pairs, in the graph that pass through *v*. For a weighted graph, the shortest path between a given pair is the sum over the weights of the edges along that path.

We find that for both the functional-inhibitory neurons and the functional-excitatory neurons that the diameter of each respective graphs increases after the cocaine injection. To be specific, the diameter of the functional-inhibitory graph is 0.31 before cocaine and 0.62 after. For the functional-excitatory neurons, the increase is even more significant with 0.46 before and 1.38 afterwards. A lower graph diameter typically correlates with a more robust graph in that it is a more tightly connected graph. Therefore, cocaine has diminished the robustness of both the functional-excitatory and functional-inhibitory network functionality. While these reported graph diameter increases are for one animal, the trend is similar for the remaining two animals. To be specific, the graph diameter of the functional-inhibitory graph is 0.18 before cocaine administration and 0.75 after, while for the functional-excitatory graph, the graph diameter goes from 0.32 to 0.39 for the second animal. For the third animal, the graph diameter of the functional-inhibitory graph is 0.24 before cocaine administration and 0.96 after; for the functional-excitatory graph, we compute a graph diameter changing from 0.46 to 1.93.

For betweenness centrality, after determining the shortest path between any two vertices, the betweenness centrality of a vertex is the number of such shortest paths that include that particular vertex. See Table 1. For the functional-excitatory effects, neurons 28 and 5 take on more of a role in the network with both of their betweenness centrality scores increasing. Both neurons are enhanced in terms of their capability to activate other neurons. Whereas for the functional-inhibitory effects, neuron 1 loses its ability to inhibit other neurons as its betweenness centrality score decreases to essentially zero, while the capability for neuron 17 (in the bottom 5 rows) to inhibit other neurons is enhanced.

**Table 1.** The betweeness centrality scores for the largest top five changes in neurons for both functional-excitatory (first five rows) and functional-inhibitory (second five rows) contributions.

Neuron	BetCent Before	BetCent After	BetCent Diff
28	0.023265	0.155510	0.132245
5	0.000000	0.102041	0.102041
17	0.000000	0.102041	0.102041
15	0.031837	0.075102	0.043265
4	0.000000	0.033878	0.033878
1	0.077551	0.000000	0.077551
17	0.000000	0.063673	0.063673
18	0.013469	0.076735	0.063265
28	0.000000	0.062857	0.062857
7	0.049796	0.000000	0.049796

### Stimulating the neuronal network

A natural question after learning the connectivity map of the neuronal network is how we can control the firing pattern. Can we send external current to the network to create a certain neuronal firing pattern that leads to a specific function in the brain?

In order to see how an input signal to the network at time *t* affects the firing pattern at time t+nδt, we start from Eq. 16 and recursively apply it to derive the following equation


(19) $$\begin{array}{rl} \vec{V}(n\;\delta t) & =T^{n}\cdot \vec{V}(0)\\ & \;+\sum_{k=0}^{n-1}T^{k}\cdot {\vec{V}}^{\left(\mathrm{ext}.\right)}\left((n-k-1)\delta t\right), \end{array}$$

where *T* is the matrix form of the connectivity matrix $T_{ij}$ without its diagonal components, and $\vec{V}=(V_{0},V_{1},\ldots )$.

**Fig. 5.**
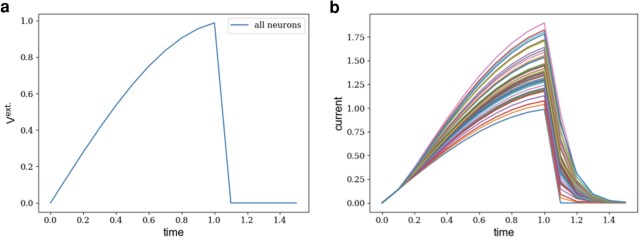
Perturbing the neuronal network. a) A time-varying external current is applied to all neurons in the neuronal network prior to cocaine administration. b) The time-varying current of all the neurons of the network induced by the same external signal. Tsame external signal induce different currents in the neurons given the functional connectivity weights. Moreover, when the external current is disconnected, all induced current begin to decay, each with their own decay rate.

We send the same time-varying external current to all of the neurons. The external current increases for a while and falls to zero at some point. The left panel of Fig. 5 depicts the time dependence of the external current. The response of the neurons to the external input is shown in the right panel of Fig. 5. An interesting observation is that while all of the neurons received the same external current, the peak of their currents is not the same. Also, the decaying rate of the neurons’ currents, after the external input is turned off, is not the same. More interestingly, the decaying rate and the peak of the currents are somewhat independent. For example, the neuron with the tallest peak decays faster than some other neurons. All of these observations can be explained by referring to the properties of the connectivity matrix $T_{ij}$. The current in the neurons is induced by (i) the external input, which is the same for all the neurons and (ii) the rest of the neurons. Therefore, the difference in the current of the neurons is a function of the signals they receive from within the network. For example, after the external current is shut down, the neurons continue to send signals to each other. Depending on their connectivity, they can receive signals for longer time, and as a result, their current decays more slowly than the rest. A similar plot is shown in Fig. S6 where two neurons are excited for a very short time and the current of the three most excited other neurons and three least excited other neurons are plotted.

### Stability analysis in the collective neuronal activity space

**Fig. 6.**
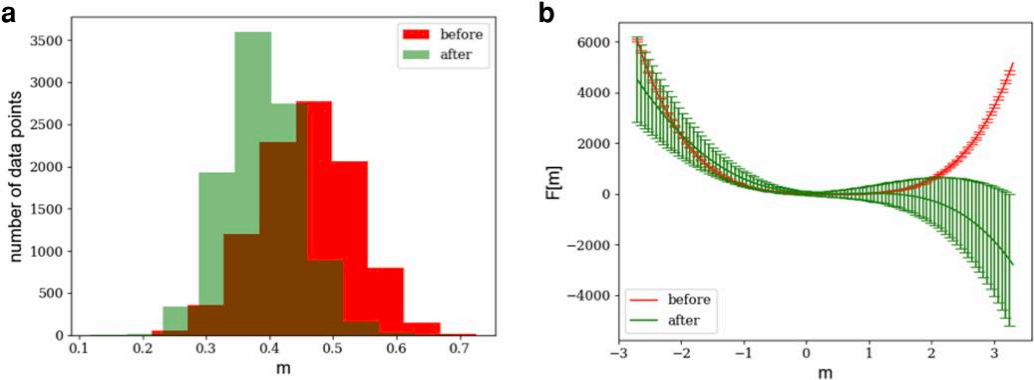
Energy functional of neuronal activity. a) Distributions of the neuronal activity *m* in before and after cocaine administration. The plot indicates the differentiation in the collective behavior of the two systems due to the variation in the experiments. In this plot, the data prior to cocaine administration is weighed such that the two histograms have the same under-the-curve area. b) Minus the logarithm of the probability function, as defined in Eq. 15, learned from data in the left panel. This plot indicates that the energy of the neuronal network prior to cocaine administration is stable where the energy has a well-defined minimum. However, following cocaine administration, the energy of the neural network is pushed to an unstable state where the probability function does not have a minimum.

While the prior subsection addresses how can use the functional connectivity to predict the firing pattern as a consequence of additional stimuli, we use our notion of energy in the collective neuronal activity space, or *m*-space, to study the changes in the parameters of the energy of Eq. 15 that are potentially induced by cocaine. First, we binarize the currents $V_{i}$ to 0 and 1 depending on whether they are below the threshold defined above. Next, we use the binary current to compute *m* at each given time. The time variation of *m* over the entire experiment, both before and after cocaine administration, is plotted in Fig. S5. The distribution of the mean-binarized-current *m* for before and after the injection is plotted in Fig. 6(left).

Our goal is to use the data in Fig. 6(left) to learn the free parameters of the energy, i.e. the parameters of the minus logarithm of the probability function. The popular method of maximum likelihood may not easily converge in this case. The reason is that the normalizing parameter $a_{0}$ in Eq. 15 is not analytically known due to the higher order terms of *m* in the probability function.

Our approach to estimate the free parameters of the probability function is to use the kernel method (58) to estimate the distribution of *m* from data in a nonparametric way. We scale the data, separately for each of the two experimental conditions, by subtracting the mean of *m* from each *m* and dividing by the standard deviation of *m*


(20) $$\begin{array}{rl} & m^{\prime }\equiv \frac{m-\mu_{m}}{\sigma_{m}},\\ & \mu_{m}=\langle m\rangle ,\\ & \sigma_{m}=\sqrt{\left\langle (m-\mu_{m}{)}^{2}\right\rangle }. \end{array}$$

Next, we use the trained estimator to predict minus the logarithm of the probability over a grid of *m*, with one million bins, that spans from −2.7 to 2.7 in the scaled space. The range is chosen such that the probability is negligible beyond it. Next, we regress the one million estimations of minus the logarithm of the probability on $\sum_{\;j=0}^{4}c_{j}m$, i.e. the Taylor expansion of the energy in the scaled space. Finally, after learning $c_{j}$ from the regression, we convert back to the nonscaled *m* space to find the values of $a_{j}$ in Eq. 15. The learned parameters are shown in Table 2.

**Table 2.** The learned parameters for before and after cocaine administration.

	$\mu_{m}$	$\sigma_{m}$	$c_{0}$	$c_{1}$	$c_{2}$	$c_{3}$	$c_{4}$
Before	0.46	0.08	1.04	− 0.04	0.38	0.01	0.002
After	0.39	0.06	0.92	0.10	0.52	− 0.03	− 0.0001

**Fig. 7.**
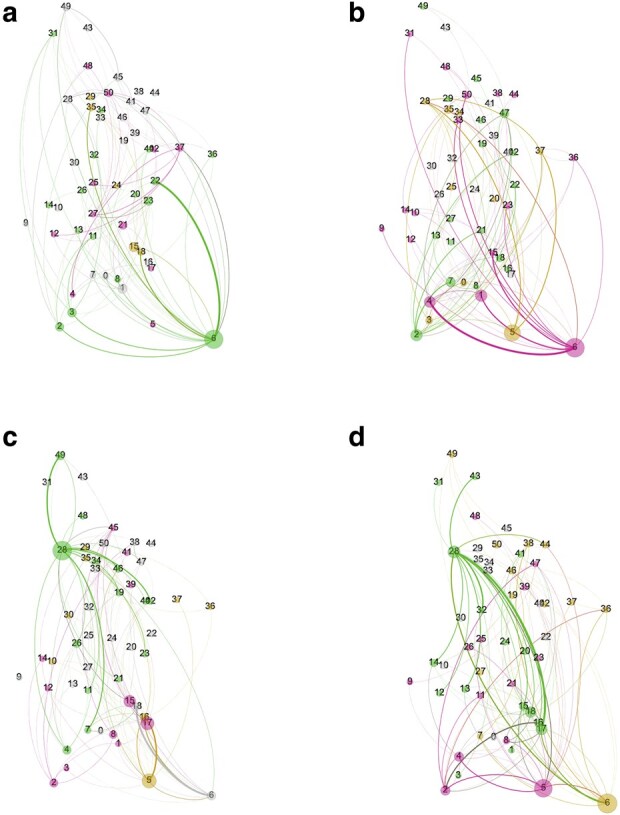
The inferred functional-excitatory and functional-inhibitory neuronal networks prior and post cocaine administration. a) and b) Networks represent prior to cocaine administration. c) and d) networks represent post cocaine administration. a) and c) Networks represent the functional-excitatory connections, while b) and d) networks represent the functional-inhibitory connections. The colors define the modularity classes, or clusters of closely interconnected nodes, using a community-finding algorithm outlined in Ref. (59) and implemented in Gephi (60). Moreover, the node sizes show the betweenness centralities. The interaction strengths are represented by the width of the edges.

We use the learned parameters $a_{j}$ and Eq. 15 to find minus the logarithm of the probability states of *m* in before and after the injection. The results, shown in Fig. 6(right), indicate that the energy of the neural network is at its minimum prior to cocaine administration. After cocaine administration, the system is shifted to a state whose energy does not have a minimum. Therefore, prior to cocaine administration, the state of the neuronal network is stable. Soon after cocaine administration, the neuronal network is in an unstable state and may presumably evolve to go back to the stable state over time as effects of the cocaine subside. Hence, the connectivity matrix of Fig. 3(right), and its representation in Fig. 7(bottom), are transient in nature. We observe a similar destabilization of the energy in a second animal in response to cocaine (see Fig. S7). In a third animal, we observe an enhancement of destabilization in the energy (see Fig. S8).

Another interesting characteristic of Fig. 6(right) is that the energy post cocaine administration drops toward positive *m*, i.e. the probability of that direction is higher. Therefore, the system will evolve toward positive *m* direction. On the other hand, in Fig. 6(left), the mean of the prior to cocaine administration is to the right of the mean of before the injection data. Putting the pieces together, after cocaine administration, its neuronal network will presumably evolve back towards prior to cocaine adminstration *m* space. Finally, the shift to the left in *m* after cocaine suggests an overall average decrease in activity as consequence of cocaine.

## Discussion

We present analysis of neuronal activity recordings from a subset of neurons in the medial prefrontal cortex of rats prior to and postadministration of cocaine. Using an underlying modern Hopfield model as a description for the neuronal network, we use a machine learning approach to determine the underlying functional connectivity of the neuronal network. The functional connectivity specifies which derived neuronal connections are functional-excitatory and which neuronal connections are functional-inhibitory as well as the strength of the functional-excitatory or functional-inhibitory interactions. We find that the functional connectivity changes after the administration of cocaine with both functional-excitatory and functional-inhibitory neurons being affected. With such quantification, we can make predictions about the neuronal network, at least over some time period, in response to external stimulation of individual neurons or even collections of them.

To explore the impact of the changes in functional connectivity in response to cocaine, there are a number of different analyses one can perform. We perform conventional network analysis in terms of unweighted topological measures of the graph. We find that the diameter of the graph increases with cocaine, suggesting that the neuronal network is less robust. We also find that the betweenness centrality scores for a few of both the functional-excitatory and functional-inhibitory neurons decrease significantly, while other scores remain essentially unchanged. Since betweenness centrality (for neurons) is a measure of the network throughput, the smaller the betweenness centrality on average, the more robust the network given that the shortest paths are more evenly distributed throughout the network as opposed to relying on a few nodes. The increase in betweenness centrality suggests that, again, the network has become less robust.

And, yet, while these measurements further quantify changes in the neuronal network in terms of graph theoretic measures, the analysis does not take into account individual neuronal activity directly. There exist methods to extract the functionality of the neuronal network, such as the controllability of neuronal networks (61). Since controllability analysis is more detailed with its various assumptions, we present a statistical analysis directly rooted in the activity of the network. Specifically, we have studied the spatially averaged neuronal activity over time and studied its statistics, just in the manner one would do with a spin system in which the sum of the neural activity relates to an order parameter of the spin system. Note that we are focusing on the neuronal excitation of the system, or the activation, and not the inhibition, as functional-inhibitory signals received by individual neurons cannot be measured with GCaMP. Just as in physical systems, a nonequilibrium version of a Landau–Ginsburg-like theory. The shape of energy functional in *m*-space, as we denote it, tells one something rather important about the state space of the system.

Our *m*-space analysis demonstrates that prior to cocaine administration, the neuronal network is stable in an energetic sense, while post cocaine administration, the neuronal network is unstable energetically. This discovery demonstrates that the administration of cocaine has strongly influenced the network to position it to be heavily influenced by other factors to be driven to a completely new stable state. In the language of substance abuse, the system is being readily driven to a new stable state that may underlie future dependence. While in these experiments, with one administration of cocaine, we anticipate the neuronal network to revert back to its initial state. With additional, consistent cocaine administration over time, we anticipate the network being driven to a new stable state becomes permanent. While experimenter-administered cocaine has been shown to produce different behavioral and neurobiological changes in animals compared to self-administration paradigms, this work is a crucial step in understanding *initial* neuronal network changes in response to cocaine.

Finally, how do our results help bring quantification to the current theories of cocaine affecting the brain. As the medial prefrontal cortex inhibits risky behavior in a top-down manner, one may be concerned with how functionality of the medial prefrontal cortex is altered to be able to “apply the brakes,” so to speak, on other brain regions affecting behavior. Given our mesoscale experiments and analysis, it is still not clear how the functionality of the medial prefrontal cortex is altered, as this neuronal network is embedded in a sea of other neurons. Shutting down, or inhibiting, the functionality of the medial prefrontal cortex seems to be the intuitive stance and supported by physiological measurements at the brain region scale (15, 16). We do indeed observe a partial shutting down, if you will, with a decrease in the neural activity amongst this neuronal subnetwork within the prefrontal cortex. However, as we observe changes in both functional-excitatory and functional-inhibitory functionality at the individual neuron scale to arrive at different neuronal firing patterns in the presence of stimulation from external, or hidden, neurons. In other words, how changes at this mesoscale affect neuronal functionality at the larger brain region scale and then between brain regions must be understood, not only for cocaine, but for other disease states as well. To address this need, we incorporated the modern Hopfield model with high quality in vivo single neuron calcium imaging in a freely behaving rat. The development of technical and computational tools for neuroscience, such as the one we have described here, contributes to our ability to identify therapeutics that are able to restore dysregulated neuronal network function and advance human health.

## Supplementary Material

pgae092_Supplementary_Data

## Data Availability

The codes and data used in this study are publicly available at https://github.com/compu-flair/BrainConnectivityAndStability.git The repository contains the scripts for data processing, analysis, and visualization, as well as the raw data files.

## References

[1] Reiner O , *et al*. 1993. Isolation of a Miller–Dicker lissencephaly gene containing G protein *β*-subunit-like repeats. Nature. 364(6439):717–721. 8355785 10.1038/364717a0

[2] Chenn A, Walsh CA. 2002. Regulation of cerebral cortical size by control of cell cycle exit in neural precursors. Science. 297(5580):365–369. 12130776 10.1126/science.1074192

[3] Kouprina N , *et al*. 2004. Accelerated evolution of the ASPM gene controlling brain size begins prior to human brain expansion. PLoS Biol. 2(5):e126. 15045028 10.1371/journal.pbio.0020126 PMC374243

[4] Rash BG, Tomasi S, Lim HD, Suh CY, Vaccarino FM. 2013. Cortical gyrification induced by fibroblast growth factor 2 in the mouse brain. J Neurosci. 33(26):10802–10814. 23804101 10.1523/JNEUROSCI.3621-12.2013 PMC3693057

[5] Shinmyo Y , *et al*. 2022. Localized astrogenesis regulates gyrification of the cerebral cortex. Sci Adv. 8(10):eabi5209. 35275722 10.1126/sciadv.abi5209 PMC8916738

[6] Liska A , *et al*. 2018. Homozygous loss of autism-risk gene CNTNAP2 results in reduced local and long-range prefrontal functional connectivity. Cereb Cortex. 28(4):1141–1153. 28184409 10.1093/cercor/bhx022

[7] Bassett DS, Bullmore E. 2006. Small-world brain networks. Neuroscientist. 12(6):512–523. 17079517 10.1177/1073858406293182

[8] Bassett DS, Bullmore ET. 2017. Small-world brain networks revisited. Neuroscientist. 23(5):499–516. 27655008 10.1177/1073858416667720 PMC5603984

[9] Lyu C, Abbott L, Maimon G. 2022. Building an allocentric travelling direction signal via vector computation. Nature. 601(7891):92–97. 34912112 10.1038/s41586-021-04067-0 PMC11104186

[10] Presigny C, Fallani FDV. 2022. Colloquium: multiscale modeling of brain network organization. Rev Mod Phys. 94(3):031002.

[11] Srivastava P, Fotiadis P, Parkes L, Bassett DS. 2022. The expanding horizons of network neuroscience: from description to prediction and control. Neuroimage. 258:119250. 35659996 10.1016/j.neuroimage.2022.119250 PMC11164099

[12] Ghosh KK , *et al*. 2011. Miniaturized integration of a fluorescence microscope. Nat Methods. 8(10):871–878. 21909102 10.1038/nmeth.1694 PMC3810311

[13] Adinoff B . 2004. Neurobiologic processes in drug reward and addiction. Harv Rev Psychiatry. 12(6):305–320. 15764467 10.1080/10673220490910844 PMC1920543

[14] Nestler EJ . 2005. The neurobiology of cocaine addiction. Sci Pract Perspect. 3(1):4–10. 18552739 10.1151/spp05314 PMC2851032

[15] Volkow ND, Michaelides M, Baler R. 2019. The neuroscience of drug reward and addiction. Physiol Rev. 99(4):2115–2140. 31507244 10.1152/physrev.00014.2018 PMC6890985

[16] Fein G, Di Sclafani V, Meyerhoff DJ. 2002. Prefrontal cortical volume reduction associated with frontal cortex function deficit in 6-week abstinent crack-cocaine dependent men. Drug Alcohol Depend. 68(1):87–93. 12167554 10.1016/s0376-8716(02)00110-2 PMC2857690

[17] Trantham-Davidson H, Lavin A. 2004. Acute cocaine administration depresses cortical activity. Neuropsychopharmacology. 29(11):2046–2051. 15138440 10.1038/sj.npp.1300482 PMC5509060

[18] Ersche KD , *et al*. 2020. Brain networks underlying vulnerability and resilience to drug addiction. Proc Natl Acad Sci U S A. 117(26):15253–15261. 32541059 10.1073/pnas.2002509117 PMC7334452

[19] DePoy LM, Gourley SL. 2015. Synaptic cytoskeletal plasticity in the prefrontal cortex following psychostimulant exposure. Traffic. 16(9):919–940. 25951902 10.1111/tra.12295 PMC4849269

[20] Shen H-W, Gipson CD, Huits M, Kalivas PW. 2014. Prelimbic cortex and ventral tegmental area modulate synaptic plasticity differentially in nucleus accumbens during cocaine-reinstated drug seeking. Neuropsychopharmacology. 39(5):1169–1177. 24232172 10.1038/npp.2013.318 PMC3957111

[21] Sequeira MK, Swanson AM, Kietzman HW, Gourley SL. 2023. Cocaine and habit training cause dendritic spine rearrangement in the prelimbic cortex. Iscience. 26(4):106240. 37153443 10.1016/j.isci.2023.106240 PMC10156587

[22] Kufahl PR , *et al*. 2005. Neural responses to acute cocaine administration in the human brain detected by fMRI. Neuroimage. 28(4):904–914. 16061398 10.1016/j.neuroimage.2005.06.039

[23] Lu H , *et al*. 2012. fMRI response in the medial prefrontal cortex predicts cocaine but not sucrose self-administration history. Neuroimage. 62(3):1857–1866. 22664568 10.1016/j.neuroimage.2012.05.076 PMC3875563

[24] Lissek T , *et al*. 2021. Npas4 regulates medium spiny neuron physiology and gates cocaine-induced hyperlocomotion. EMBO Rep. 22(12):e51882. 34661342 10.15252/embr.202051882 PMC8647009

[25] Hopfield JJ . 1982. Neural networks and physical systems with emergent collective computational abilities. Proc Natl Acad Sci. 79(8):2554–2558. 6953413 10.1073/pnas.79.8.2554 PMC346238

[26] Krotov D, Hopfield J. 2020. Large associative memory problem in neurobiology and machine learning, arXiv, arXiv:2008.06996, preprint: not peer reviewed.

[27] Bassett DS, Sporns O. 2017. Network neuroscience. Nat Neurosci. 20(3):353–364. 28230844 10.1038/nn.4502 PMC5485642

[28] Bassett DS, Zurn P, Gold JI. 2018. On the nature and use of models in network neuroscience. Nat Rev Neurosci. 19(9):566–578. 30002509 10.1038/s41583-018-0038-8 PMC6466618

[29] Van Essen DC , *et al*. 2012. The human connectome project: a data acquisition perspective. Neuroimage. 62(4):2222–2231. 22366334 10.1016/j.neuroimage.2012.02.018 PMC3606888

[30] Messé A, Hütt M-T, König P, Hilgetag CC. 2015. A closer look at the apparent correlation of structural and functional connectivity in excitable neural networks. Sci Rep. 5:1. 10.1038/srep07870 PMC4297952 25598302

[31] Wang L, Lin FV, Cole M, Zhang Z. 2021. Learning clique subgraphs in structural brain network classification with application to crystallized cognition. NeuroImage. 225:117493. 33127479 10.1016/j.neuroimage.2020.117493 PMC7826449

[32] Amit DJ, Gutfreund H, Sompolinsky H. 1987. Statistical mechanics of neural networks near saturation. Ann Phys (N Y). 173(1):30–67.

[33] Tkačik G , *et al*. 2013. The simplest maximum entropy model for collective behavior in a neural network. J Stat Mech: Theory Exp. 2013(03):P03011.

[34] Mora T, Bialek W. 2011. Are biological systems poised at criticality? J Stat Phys. 144(2):268–302.

[35] Helias M, Dahmen D. 2020. Statistical field theory for neural networks. Vol. 970. Cham, Switzerland: Springer International Publishing.

[36] Segadlo K , *et al*. 2022. Unified field theoretical approach to deep and recurrent neuronal networks. J Stat Mech: Theory Exp. 2022(10):103401.

[37] Di Santo S, Villegas P, Burioni R, Muñoz MA. 2018. Landau–Ginzburg theory of cortex dynamics: scale-free avalanches emerge at the edge of synchronization. Proc Natl Acad Sci U S A. 115(7):E1356. 29378970 10.1073/pnas.1712989115 PMC5816155

[38] Zhou P , *et al*. 2018. Efficient and accurate extraction of in vivo calcium signals from microendoscopic video data. elife. 7:e28728. 29469809 10.7554/eLife.28728 PMC5871355

[39] Hopfield JJ, Tank DW. 1986. Computing with neural circuits: a model. Science. 233(4764):625–633. 3755256 10.1126/science.3755256

[40] Burkitt AN . 2006. A review of the integrate-and-fire neuron model: I. Homogeneous synaptic input. Biol Cybern. 95(1):1–19. 16622699 10.1007/s00422-006-0068-6

[41] Amit DJ, Gutfreund H, Sompolinsky H. 1985. Storing infinite numbers of patterns in a spin-glass model of neural networks. Phys Rev Lett. 55(14):1530–1533. 10031847 10.1103/PhysRevLett.55.1530

[42] Yan H , *et al*. 2013. Nonequilibrium landscape theory of neural networks. Proc Natl Acad Sci U S A. 110:E4185. 24145451 10.1073/pnas.1310692110 PMC3831465

[43] Zhong W, Lu Z, Schwab DJ, Murugan A. 2020. Nonequilibrium statistical mechanics of continuous attractors. Neural Comput. 32(6):1033–1068. 32343645 10.1162/neco_a_01280

[44] Jaynes ET . 1957. Information theory and statistical mechanics. Phys Rev. 106(4):620–630.

[45] Jaynes ET . 1957. Information theory and statistical mechanics. II. Phys Rev. 108(2):171–190.

[46] Schneidman E, Berry MJ, Segev R, Bialek W. 2006. Weak pairwise correlations imply strongly correlated network states in a neural population. Nature. 440(7087):1007–1012. 16625187 10.1038/nature04701 PMC1785327

[47] Gu S , *et al*. 2018. The energy landscape of neurophysiological activity implicit in brain network structure. Sci Rep. 8(1):2507. 29410486 10.1038/s41598-018-20123-8 PMC5802783

[48] Wilson HR, Cowan JD. 1972. Excitatory and inhibitory interactions in localized populations of model neurons. Biophys J. 12(1):1–24. 4332108 10.1016/S0006-3495(72)86068-5 PMC1484078

[49] Wilson HR, Cowan JD. 2021. Evolution of the Wilson–Cowan equations. Biol Cybern. 115(6):643–653. 34797411 10.1007/s00422-021-00912-7

[50] Radhiyanti PT, Konno A, Matsuzaki Y, Hirai H. 2021. Comparative study of neuron-specific promoters in mouse brain transduced by intravenously administered AAV-PHP. eB. Neurosci Lett. 756:135956. 33989730 10.1016/j.neulet.2021.135956

[51] Veres JM, Andrasi T, Nagy-Pal P, Hajos N. 2023. Camkii*α* promoter-controlled circuit manipulations target both pyramidal cells and inhibitory interneurons in cortical networks. Eneuro. 10(4):ENEURO.0070-23.2023. 10.1523/ENEURO.0070-23.2023 PMC10088982 36963833

[52] Pedregosa F , *et al*. 2011. Scikit-learn: machine learning in Python. J Mach Learn Res. 12:2825.

[53] Winer J, Larue D. 1989. Populations of GABAergic neurons and axons in layer I of rat auditory cortex. Neuroscience. 33(3):499–515. 2636704 10.1016/0306-4522(89)90402-8

[54] Alreja A, Nemenman I, Rozell CJ. 2022. Constrained brain volume in an efficient coding model explains the fraction of excitatory and inhibitory neurons in sensory cortices. PLoS Comput Biol. 18(1):e1009642. 35061666 10.1371/journal.pcbi.1009642 PMC8809590

[55] Honey CJ, Kötter R, Breakspear M, Sporns O. 2007. Network structure of cerebral cortex shapes functional connectivity on multiple time scales. Proc Natl Acad Sci U S A. 104(24):10240–10245. 17548818 10.1073/pnas.0701519104 PMC1891224

[56] Crisanti A, Sompolinsky H. 1987. Dynamics of spin systems with randomly asymmetric bonds: Langevin dynamics and a spherical model. Phys Rev A. 36(10)4922–4939. 10.1103/physreva.36.4922 9898751

[57] Xu P, Chen A, Li Y, Xing X, Lu H. 2019. Medial prefrontal cortex in neurological diseases. Physiol Genom. 51(9):432–442. 10.1152/physiolgenomics.00006.2019 PMC6766703 31373533

[58] Borzou A, Patteson AE, Schwarz JM. 2021. A data-driven statistical description for the hydrodynamics of active matter. New J Phys. 23(10):103004.

[59] Blondel VD, Guillaume J-L, Lambiotte R, Lefebvre E. 2008. Fast unfolding of communities in large networks. J Stat Mech: Theory Exp. 2008(10):P10008.

[60] Bastian M, Heymann S, Jacomy M. 2009. Gephi: an open source software for exploring and manipulating networks. Proceedings of the International AAAI Conference on Web and Social Media. 3(1):361–362. 10.1609/icwsm.v3i1.13937.

[61] Gu S , *et al*. 2015. Controllability of structural brain networks. Nat Commun. 6(1):8414. 26423222 10.1038/ncomms9414 PMC4600713

