# Correction to: Cocaine diminishes functional network robustness and destabilizes the energy landscape of neuronal activity in the medial prefrontal cortex

**DOI:** 10.1093/pnasnexus/pgaf257

**Published:** 2025-09-24

This is a correction to: Ahmad Borzou, Sierra N Miller, Jonathan D Hommel, J M Schwarz, Cocaine diminishes functional network robustness and destabilizes the energy landscape of neuronal activity in the medial prefrontal cortex, *PNAS Nexus*, Volume 3, Issue 3, March 2024, pgae092, https://doi.org/10.1093/pnasnexus/pgae092

The Funding section of this manuscript has been updated to correct the following sentence:

“J.M.S. wishes to acknowledge funding from NSF-DMR-2204312.”

The revised sentence reads:

“J.M.S. acknowledges funding support from the National Science Foundation via DMR-2204312.”

