# Supplementary material for: Cocaine diminishes functional network robustness and destabilizes the energy landscape of neuronal activity in the medial prefrontal cortex
Source: PNAS Nexus. 2024 Mar 6;3(3):pgae092. doi: 10.1093/pnasnexus/pgae092 (PMC10929585; doi:10.1093/pnasnexus/pgae092)
Supplement: pgae092_Supplementary_Data [file pgae092_supplementary_data.pdf]

Supplemental Material: Cocaine diminishes functional network robustness and destabilizes the energy landscape of neuronal activity in the medial prefrontal cortex

Ahmad Borzou, Sierra N. Miller, Jonathan D. Hommel, J. M. Schwarz

February 19, 2024

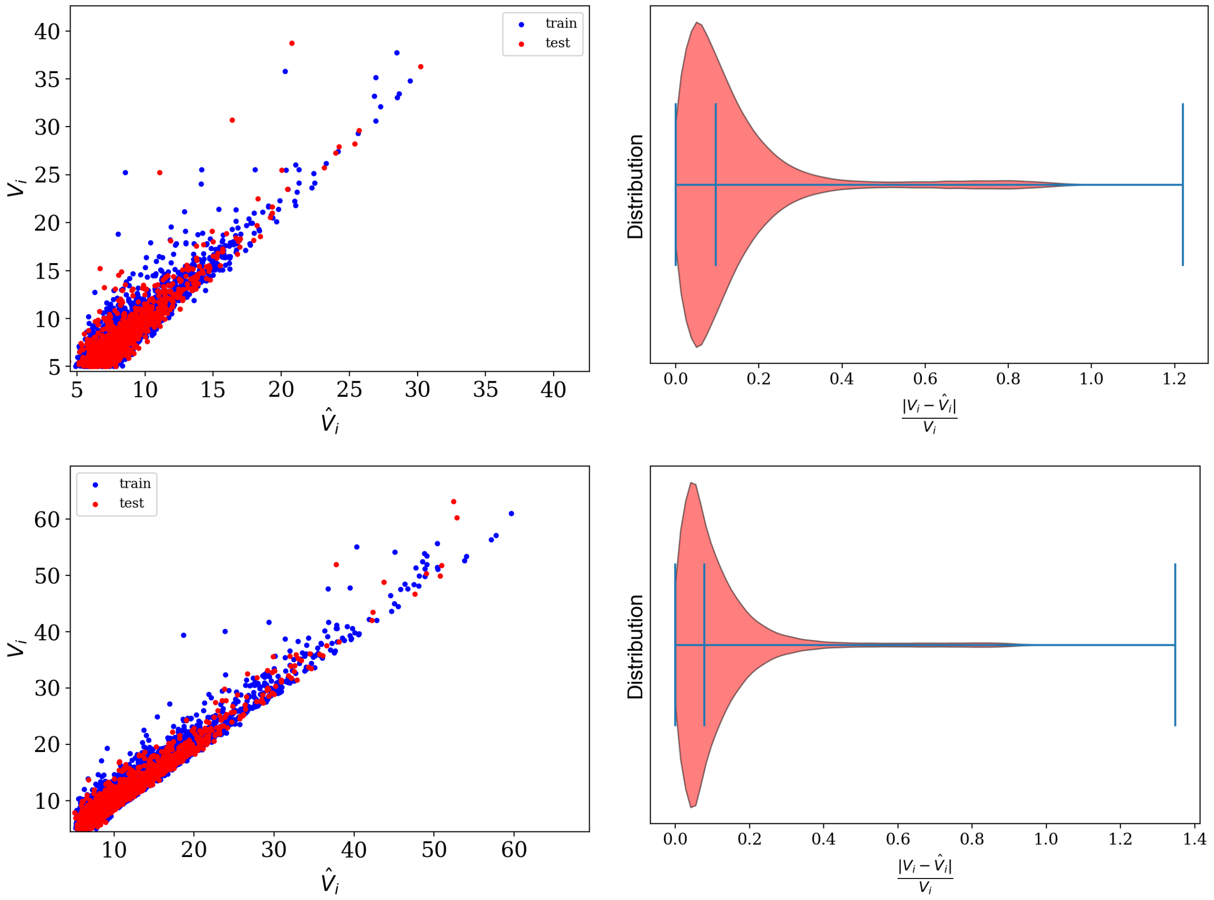

Figure S1: *Learning the parameters.* This figure shows the results of fitting Eq.16 to estimate the parameters in T_{ij} and $V_i^{(\text{ext.})}$.. Top: Prior cocaine administration. Bottom: Post cocaine administration. Left: the forecasting prediction versus the true value in the first neuron of the set. Right: the violin plot of the error, as defined in the x label, of all the neurons and all the data points in the test set. The first and last vertical lines show where the 0%, and 100% of the data is located. The middle vertical line represents the median. The agreement between the predicted and observed neural activities suggests the reliability of the estimated parameters.

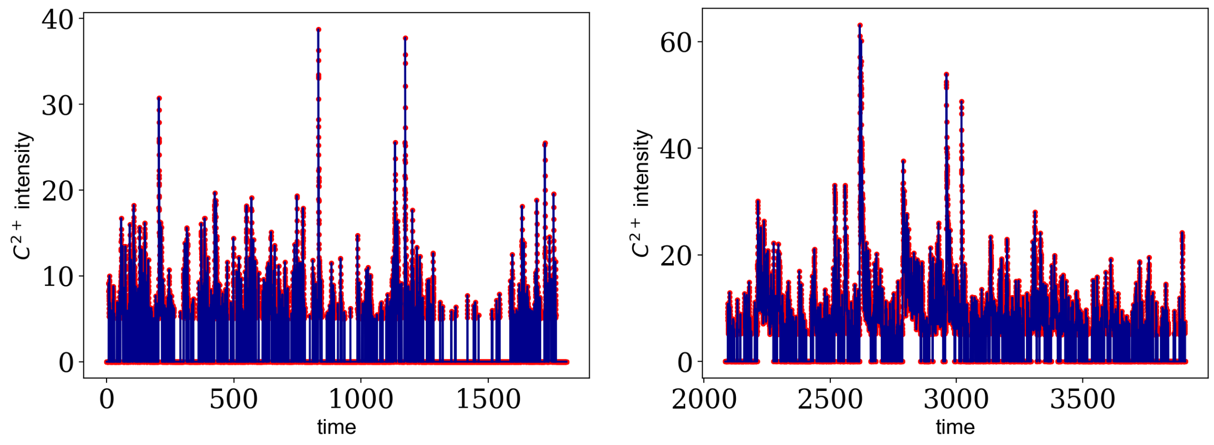

Figure S2: *Relative calcium ion intensity in the first neuron over time.* Each red dot indicates one data point. Left: Prior to cocaine administration. Right: Post cocaine administration.

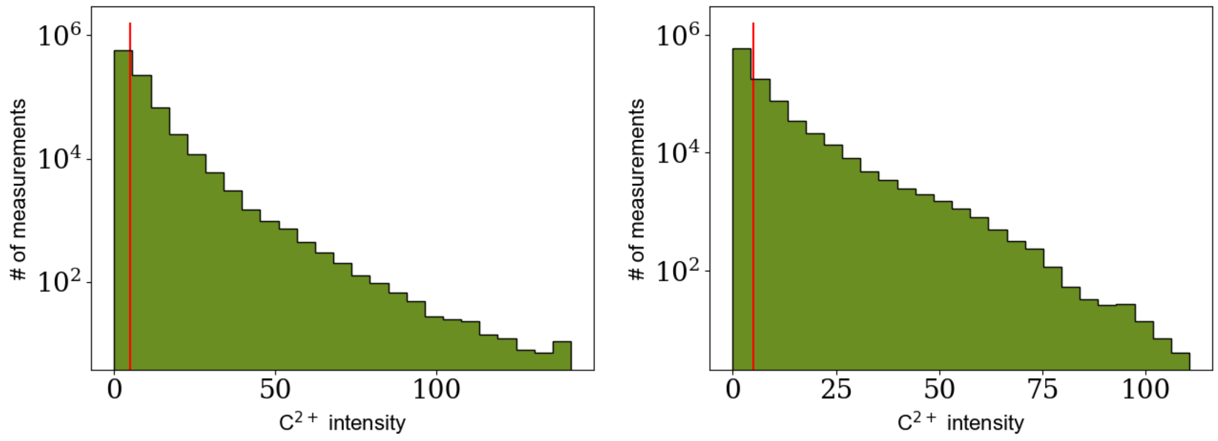

Figure S3: *Distribution of relative calcium ion intensity for all neurons over the course of the experiment.* Left: Prior to cocaine administration. Right: Post cocaine administration. The relative intensities to the left of the red vertical lines are considered noise and set to zero.

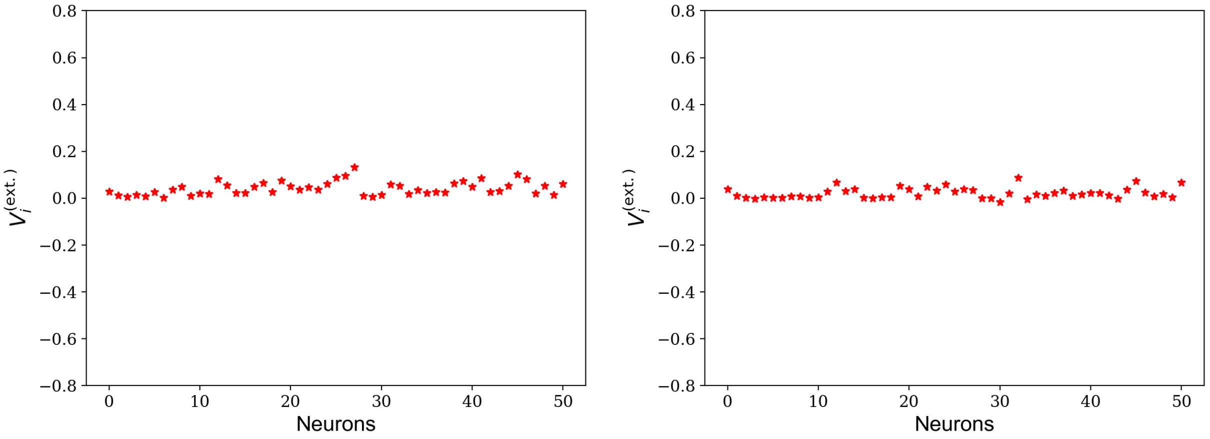

Figure S4: *External input to neurons from either hidden neurons or direct stimulus.* Left: Prior to cocaine administration. Right: Post cocaine administration. The external inputs are negligible in comparison to the connectivity matrices.

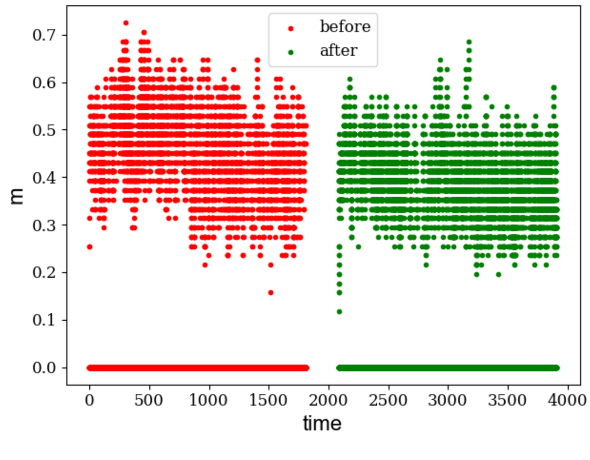

Figure S5: *Variation of the average neural current m with time.* Both before and after cocaine administration data are shown with their corresponding timings.

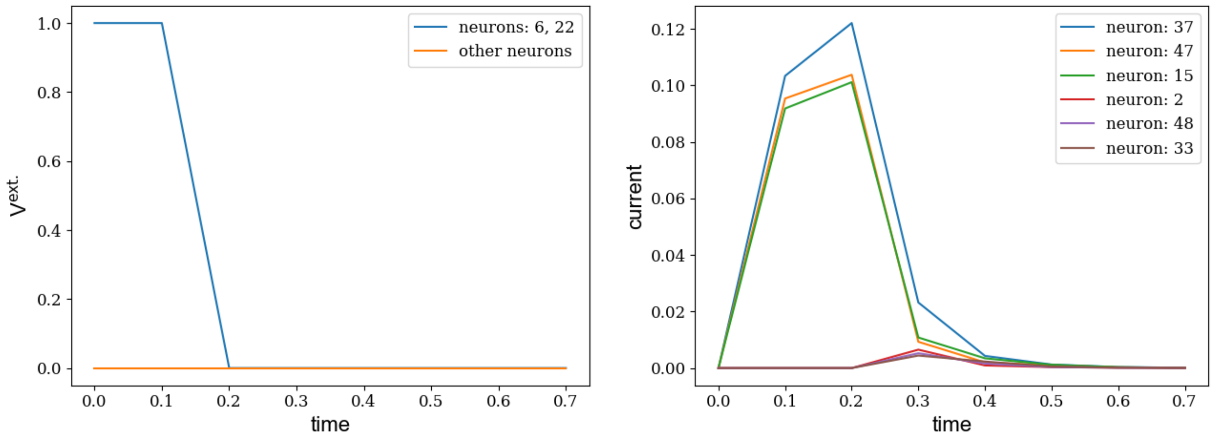

Figure S6: *More perturbations to the neuronal network.* Left: the time-varying external current applied to the neurons in a prior-cocaine-administration network. Only neurons 6 and 22 have received a fast decaying stimulus. Right: The time-varying currents induced by the external signal in the three most excited and three least excited neurons, other than the stimulated ones. Interestingly, the peak of the excited neurons is one time step ahead of when the external input is disconnected. Moreover, the three least excited neurons are not excited right away. When the three most excited neurons are discharging, the least excited neurons start to excite. These are the effects of the connectivity matrix.

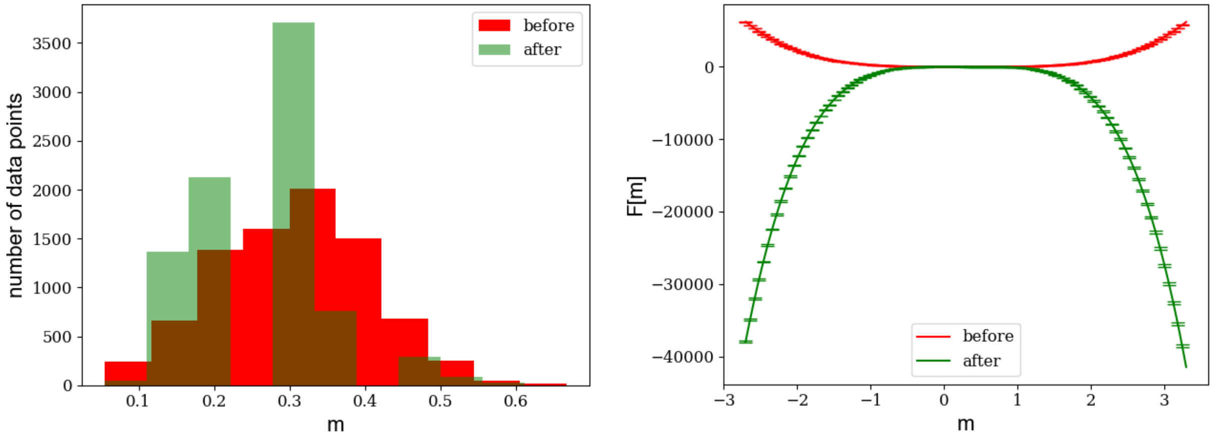

Figure S7: *Destabilization of the energy in response to cocaine administration.* Left: Distributions of the neuronal activity m before and after cocaine administration for a second rat. The plot indicates the differentiation in the collective behavior due to cocaine administratoin. The data prior to cocaine is weighed such that the two histograms have the same under-the-curve area. Right: Minus the logarithm of the probability function, as defined in Eq. 15, learned from the data in the left panel. This plot indicates that the neural network energy is stable where the energy has a well defined minimum. However, after cocaine administration, the neural network is pushed to an unstable state where the probability function does not have a maximum.

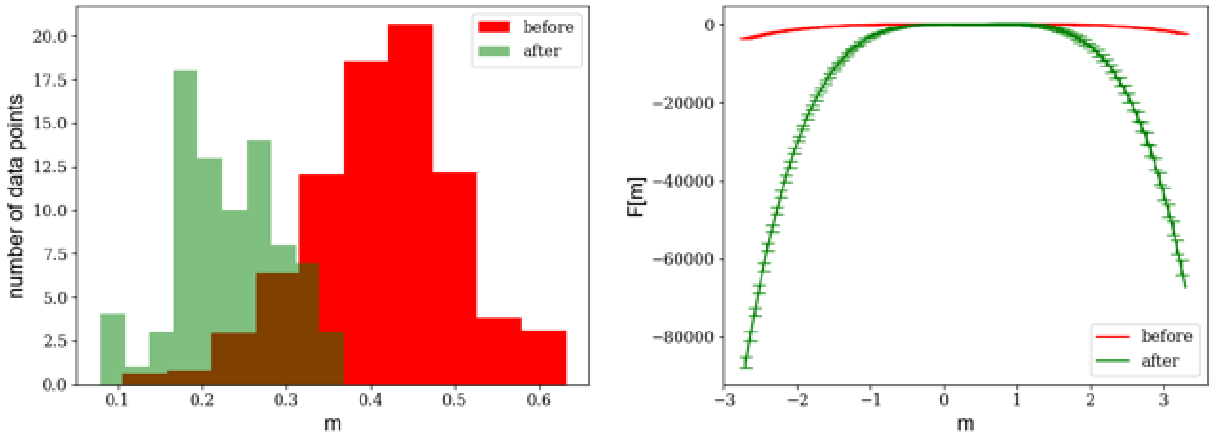

Figure S8: *Enhanced destabilization of the energy in response to cocaine administration.* Left: Distributions of the neuronal activity m before and after cocaine administration for a third rat. The plot indicates the differentiation in the collective behavior due to cocaine administration. The data prior to cocaine is weighed such that the two histograms have the same under-the-curve area. Right: Minus the logarithm of the probability function, as defined in Eq. 15, learned from the data in the left panel. This plot indicates that the neural network energy is not stable for the third animal both before and after cocaine administration, the instability of the free energy landscrape is enhanced after cocaine administration.

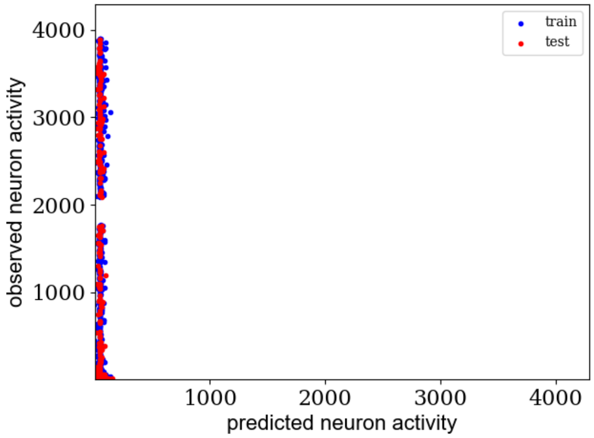

Figure S9: *Randomly shuffled data as a null hypothesis.* The result of autoregression time series forecasting on the shuffled dataset for the first neuron in the set. The shuffled dataset plays the roll of a null hypothesis. This plot indicates the significance of the connectivity matrix presented in Fig. 3.
